# Assessment of Immunogenicity of Adjuvanted Quadrivalent Inactivated Influenza Vaccine in Healthy People and Patients With Common Variable Immune Deficiency

**DOI:** 10.3389/fimmu.2020.01876

**Published:** 2020-08-19

**Authors:** Aristitsa Mikhailovna Kostinova, Nelli Kimovna Akhmatova, Elena Alexandrovna Latysheva, Yulia Alexeevna Dagil, Svetlana Valentinovna Klimova, Anna Egorovna Vlasenko, Ekaterina Alexandrovna Khromova, Tatyana Vasilievna Latysheva, Mikhail Petrovich Kostinov

**Affiliations:** ^1^National Research Center Institute of Immunology Federal Medical-Biological Agency of Russia, Moscow, Russia; ^2^Federal State Budgetary Scientific Institution I. Mechnikov Research Institute of Vaccines and Sera, Moscow, Russia; ^3^Novokuznetsk State Institute for Advanced Training of Physicians, Branch Campus of the Russian Medical Academy of Continuous Professional Education, Novokuznetsk, Russia

**Keywords:** adjuvanted QIV, immunogenicity, influenza, vaccination, CVID, azoximer bromide

## Abstract

**Background:** Recent addition to vaccines of adjuvants has been actively used to enhance the immunogenicity. However, the use of adjuvants for the development of quadrivalent inactivated influenza vaccines (QIV) is currently limited. The aim of this study was to examine immunogenicity of adjuvanted QIV in healthy people and patients with primary immune deficiency—common variable immune deficiency (CVID).

**Methods:** In total before the flu season 2018–2019 in the study were involved 32 healthy volunteers aged 18–52 years and 6 patients with a confirmed diagnosis of CVID aged 18–45 years. To evaluate antibody titers 21 days after vaccination against the influenza A and B strains a hemagglutination inhibition assay (HI) was used.

**Results:** In healthy volunteers adjuvanted QIV has proved its immunogenicity to strains A/H1N1, A/H3N2, B/Phuket and B/Colorado in seroprotection (90, 97, 86, and 66%, respectively), seroconversion (50, 60, 52, and 45%, respectively), GMR (6.2, 5.7, 4.2, and 3.4, respectively). Statistically significant differences in the level of all criteria were revealed between groups of healthy and CVID patients regardless of the virus strain. Most patients with CVID showed an increase in post-vaccination antibody titer without reaching conditionally protective antibody levels.

**Conclusion:** Immunization with single dose of adjuvanted QIV with decreased amount of hemagglutinin protein to all virus strains due to the use of azoximer bromide forms protective immunity in healthy people, but in patients with CVID the search for new vaccination schemes is the subject of further investigations, as well as the effectiveness of boosterization with adjuvant vaccines.

## Introduction

Influenza virus infection, caused by single-stranded RNA viruses belonging to the Orthomyxoviridae family, is associated with significant morbidity and mortality worldwide, and affects particularly risk groups such as patients with cardiopulmonary conditions, pregnant women and children, old people and immunocompromised patients. It impacts all countries: every year, there are an estimated 1 billion cases, 3–5 million severe cases, and 290–650 000 influenza-related respiratory deaths worldwide (1).

The first vaccine against the influenza virus was created in 1944, included two strains of the influenza virus until in 1978 was developed the first trivalent both inactivated (TIV) and live attenuated influenza vaccine, which was broadly used for immunization (2). The vaccine included two strains of type A influenza virus and one of two genetically distinct type B influenza lineages (Yamagata or Victoria) which WHO annually choose for inclusion in formulation of influenza vaccines in Northern and Southern hemispheres (3). However, an analysis over 10 years in the USA and 8 years in Europe showed a mismatch between the circulating in population seasonal lineage and the vaccine Lineage of type B influenza virus in 25–50% seasons from 2001to 2011 years of analysis (4, 5). The same situation was seen in the Russian Federation in the period from 2006 to 2015, when the mismatch was found in 3 of 9 seasons (6). That is why in 2012 WHO recommended for use new inactivated quadrivalent influenza vaccines which include both B lineages besides both A strains.

Two modeling studies performed in the USA and Germany concluded that QIV could have prevented ~395,000 infections per year in the world and at least 30,000 cases, 3,500 hospitalizations, and 700 deaths in the USA population caused by B lineage mismatch (7, 8).

In numerous studies conducted both at the preclinical stage and already in vaccinated adults, inactivated QIV was as immunogenic as seasonal TIV, with equivalent efficacy against the shared three strains included in TIV, and a superior immunogenicity against the non-TIV B lineage (9).

In recent decades, addition to vaccines of adjuvants, that allow to reduce the amount of included antigens with the level of post-vaccination IgG which are synthesized in a short time at the same or even higher level than after non-adjuvant vaccines, have been used to enhance the immunogenicity. However, the use of adjuvants for the development of QIV is currently limited.

Adjuvant is a non-specific immunostimulant of inorganic and organic genesis, which increases the specific immune response to antigens. They have been used for over 90 years and currently are the components of more than 30 licensed vaccines, among them influenza vaccines from different manufacturers (10). The inclusion of an adjuvant allows to reduce the amount of virus antigen and the number of immunizations (doses) to create a stable immunity to infectious diseases. For example, in the UK, an influenza vaccine containing 15 strains of the virus is currently being developed, while the dose of antigen in it is reduced by 100 times, due to the remaining danger of a pandemic, according to the WHO (11). Despite different mechanism, almost all adjuvants initially influence on antigen-presenting cell (12, 13). In addition, some of them are able to interact with B-lymphocytes, that also leads to stimulation of the humoral immunity.

In studies conducted in Russia devoted to a trivalent subunit polymer (immuno-adjuvant) influenza vaccine containing 5 μg of antigens of two virus strains type A and one virus strains type B, and azoximer bromide used as an adjuvant, it was shown that specific antibodies were synthesized in values similar to subunit non-adjuvant vaccines (14). It can be assumed that when using the same amount of adjuvant (500 μg of azoximer bromide), but with an increased number of different virus antigens, a similar effect will be obtained.

It is especially important to achieve protection against influenza in patients with defects in the humoral immunity, who respond with a low level of specific antibodies or lack of their synthesis after vaccination. It should be noted that one of the criteria for the diagnosis of common variable immune deficiency (CVID) in a group of patients with primary immunodeficiency (PID) with defects predominantly in the humoral immunity is a poor antibody response to vaccines, i.e., absence of protective levels despite vaccination (15).

To our knowledge, currently only three studies have examined the formation of post-vaccination immune response in a limited number of CVID patients, where are reported data on their ability to synthesize specific antibodies and induce cell immunity in response to influenza vaccines (16–18). Two of them were conducted with the use of adjuvanted influenza pandemic vaccine A/California/7/2009 (H1N1)-like split virus (X179a) adjuvanted with the oil-in-water emulsion AS03. In the study of Pedersen et al. the number of participants with CVID was only 3, while the author reported that two of them responded to the vaccination by a >4-fold rise in haemagglutination inhibition (HI) antibodies (19). In another study of the same vaccine, published in 2018, 48 CVID patients were vaccinated against influenza, and it was detected that 8 (16.7%) patients had reached a ≥1:40 titer of specific antibodies against the pandemic influenza A(H1N1) antigen: 4 after the first vaccination, the other 4—after booster dose 1 month later (67–98.3% of healthy people form protective antibody levels since 21days after a single dose of Pandemrix®) (20). In the third study after immunization with a non-adjuvant influenza vaccine 1 of 8 responded by synthesis if antibodies against at least 1 of the 3 vaccine strains (17).

## Aim of the Study

To examine the formation of humoral immunity after immunization with the quadrivalent inactivated subunit adjuvanted influenza vaccine to virus strains in healthy people and patients with CVID.

## Materials and Methods

In an open-label, single-center, non-randomized, prospective, cohort, controlled study the effect of influenza tetravalent inactivated subunit adjuvanted vaccine on antibody synthesis in healthy volunteers and patients with CVID was examined.

### Patient Description

In total before the flu season 2018–2019 in study were enrolled 32 healthy volunteers aged 18–52 years. The comparison group consisted of 6 patients with a confirmed diagnosis of Common variable immune deficiency aged 18–45 years who met the inclusion criteria.

CVID is one of the most frequently diagnosed primary immunodeficiencies. People with CVID are highly susceptible to bacterial or more rarely viral invaders and often develop recurrent infections, particularly in the lungs, sinuses, and ears. The characteristic laboratory features include low levels of serum immunoglobulins [marked decrease of IgG and marked decrease of IgA with or without low IgM levels (measured at least twice; <2SD of the normal levels for age)], which causes an increased susceptibility to infection (18). Another part of the diagnosis of CVID is a lack of functional antibody in serum against vaccine antigens such as tetanus, diphtheria, pneumococcal polysaccharide (21). They have absence of protective levels despite vaccination. The treatment of CVID is monthly intravenous immunoglobulin (IVIG) replacement therapy during all life period. All the important IgG antibodies presented in normal population are extracted from a large pool of human plasma from donors.

### Inclusion Criteria

- Healthy volunteers aged from 18 to 52 years without chronic bronchopulmonary, cardiovascular, rheumatological diseases, hepatic or renal impairment, metabolic disorders confirmed by anamnestic data or objective clinical examination.
- Confirmed diagnosis CVID in accordance with diagnostic criteria established by the European Society for Immunodeficiency Diseases (http://esid.org/WorkingParties/Registry/Diagnosis-criteria) and the American Academy of Allergy, Asthma and Immunology for the diagnosis and treatment of PID.
- IVIG therapy no later than 28 days before vaccination and no earlier than 21 days after it, that is, a break between two subsequent administrations of immunoglobulins for at least 7 weeks.
- Signed informed consent.

### Exclusion Criteria

- Symptoms of influenza or flu-like illness in the past 6 months.
- Symptoms of acute infection at the time of vaccination and during 1 month before current vaccination.
- Glucocorticosteroid or other immunosuppressive therapy admission at the time of the study and 3 months before the start.
- Symptoms of enteropathy with protein loss in patients with CVID at the time of the study.

All participants in the previous season (2017–2018) did not receive influenza vaccine and no influenza infection was registered, although in the 2016–2017 season some of the healthy volunteers were immunized against influenza that was not observed among patients with CVID who have not been vaccinated in previous two flu seasons.

Vaccination was conducted in the Department of Immunopathology in the Institute of Immunology of the FMBA of Russia. The laboratory part of the study was carried out in the laboratory of the Mechanisms of immune regulation in Mechnikov Research Institute of Vaccines and Sera in Moscow. The study was conducted according to the Russian Federation National Standard Protocol ΓOCTP 52379-2005 Good Clinical Practice≫ and International GCP standards (22). The study was based on the ethical principles and recommendations of the WHO and the Russian Ministry of Health. All patients signed the informed consent for the participation.

### Vaccines

First immunization was carried out on the 26 of November 2018 and the last on the 21 of February 2019. Single-dose vaccination was performed according to the manufacturer's instructions. All patients received the Quadrivalent inactivated subunit adjuvanted influenza vaccine Grippol® Quadrivalent (NPO Petrovax Pharm LLC, Russia).

Grippol® Quadrivalent is the first Russian quadrivalent inactivated subunit adjuvanted influenza vaccine manufactured in Russia full-cycle starting from active pharmaceutical ingredient production to the applicable GMP regulations. Grippol Quadrivalent contained four viral strains as recommended by the WHO: A/Michigan/45/2015 (H1N1)pdm09-like virus; A/Singapore/INFIMH-16-0019/2016 (H3N2)-like virus; B/Colorado/06/2017-like virus (B/Victoria/2/87 lineage); B/Phuket/3073/2013-like virus (B/Yamagata/16/88 lineage).

The key benefit of the vaccine is a decreased amount of hemagglutinin protein due to the use of Polyoxidonium (azoximer bromide)—a water-soluble high-molecular immune system adjuvant that enhances the immune response to vaccination and provides for cutting the antigen load three-fold as compared to traditional technologies. This antigen sparing technology is unique; for more than 20 years, it has been used in Russia to produce vaccines that have been successfully administered within the framework of the national immunization schedules in the Russian Federation and other countries. In 1 vaccination dose (0.5 ml) there are 20 μg of antigens (5 μg of hemagglutinin of each strain) and 500 mcg of azoximer bromide.

### Blood Samples

Serum intake for determining the level of virus-specific antibodies was performed before vaccination, 21–22 days and 3 months after vaccination. On the 21–22 days after vaccination, the study participants in the group of patients with CVID were scheduled to undergo IVIG therapy in a standard dose of 0.4 g/kg. The next sampling of whole blood was 3 months after vaccination on the background of IVIG therapy.

### Laboratory Methods

To evaluate antibody titers against the influenza A and B strains a HI assay was used as recommended by CDC method for evaluating the immunogenicity of influenza vaccines (23). To remove non-specific inhibitors of hemagglutination, test sera were incubated at 37°C overnight (19 ± 1 h) at 1:4 dilution with receptor-destroying enzyme (RDE; Denka Seiken, Tokyo, Japan) followed by a 30-min inactivation step at 56°C and further dilution to 1:10 with phosphate-buffered saline (PBS). HI assay was performed with 0.5% chicken RBC and 4 hemagglutination units of antigens. Antigens for HI assay were provided by Smorodintsev Research Institute of Influenza (WHO National Influenza Center of Russia, Saint-Petersburg).

To determine specific antibodies were used strains, recommended by WHO for quadrivalent vaccines in 2018–2019 influenza season: A/H1N1/Michigan 45/15, A/H3N2/Singapore/INFMH-16-0019/16, B/Colorado/06/17 (B/Victoria lineage), and B/Phuket/3073/13 (B/Yamagata lineage).

To evaluate immunogenicity of the influenza vaccine according to the Guideline on clinical evaluation of vaccines of the Committee on Human Medicinal Products (CHMP) criteria for adult patients were used (24):

1. Seroprotection level—percentage of vaccinated patients with a hemagglutinin-inhibiting antibodies titer ≥1:40 on the 21 day after vaccination (reference level—over 70%).
2. Seroconversion level—percentage of vaccinated patients with either a pre-vaccination HI titer <10 and a post-vaccination HI titer ≥40 or a pre-vaccination HI titer ≥10 and a ≥4-fold increase in HI titer on the 21 day after vaccination (reference level—over 40%)
3. Geometric mean antibody titers (GMT)
4. Geometric mean ratio (GMR)—increase in the mean geometric titer of hemagglutinin-inhibiting antibodies on the 21 days after vaccination compared to baseline (reference level—over 2.5-fold).

The efficacy and immunogenicity of the vaccine is considered to be satisfactory if the vaccine meets at least one of these criteria.

### Statistical Analysis

For the intergroup comparison of qualitative signs (seroprotection and seroconversion levels) the Chi-Square test was used, in the case of cells in the table with expected frequencies of <5%, the exact Fisher test was used. Comparison of qualitative characteristics in related samples (in the dynamics between control points) was carried out using the McNemar test. Descriptive statistics of qualitative characteristics are represented by the fraction, 95% confidence interval of the fraction calculated by the Clopper-Pearson method, and the absolute number of subjects with the studied characteristic in the total number of group (n/N). Descriptive statistics of quantitative characteristics are represented by the geometric mean and its 95% confidence interval. To apply the statistical criteria the initial quantitative data were pre-logarithmized and checked for compliance with the normal distribution (the Shapiro-Wilks test was used). The check showed that all the pre-logarithmized data correspond to the normal distribution. To compare two independent groups by quantitative criteria, the Student criterion was used (in the absence of equality of variances, which was checked by the Livin test, the Student criterion with the Welch modification was used). Comparison of quantitative characteristics in related groups (in the dynamics between control points) was carried out by the Student criterion for related samples. Calculation of criteria for quantitative characteristics was carried out on logarithmized data. The analysis assumed a comparison between the values of characteristics at the control point of 1 month and the initial level and control points of 3 months and 1 month; if a statistically significant difference for 1–3 months was detected, the values of characteristics at the control point of 3 months was compared with the initial level. All calculations were carried out in a freely distributed statistical environment R (v.3.6), the “stats” package (v.3.6.2) was used.

## Results

### Seroprotection Level

**Figure 1:**
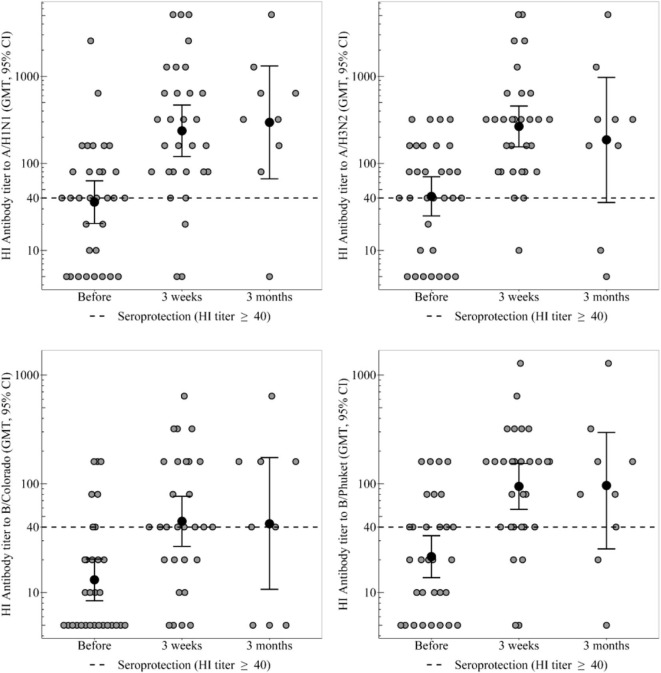
HI antibody titers, which show the individual titers of healthy participants and GMT (95% CI) in the group of healthy participants. In the group of healthy participants, a statistically significant increase in the proportion of seropositive was observed 3 weeks after immunization toward all strains. Three months after vaccination statistically significant decrease in seroprotection level was not detected for any strain. In the group of CVID patients the GMT remains unchanged throughout the whole period, regardless of any strain. In the group of healthy participants a 3 weeks after immunization statistically significant increase in antibody titer was observed for all virus strains.

Analysis of the results with assessment of the seroprotection level in the groups of healthy participants and CVID patients is presented in Table 1 and Figure 1.

**Table 1:** Seroprotection level in the groups of healthy participants and patients with CVID.

**Virus strain**	**Period**	**Healthy participants (*****n*** **= 32)**			**Patients with CVID (n = 6)**			**Between groups^a^**
		**People**	**%**	**95%CI**	**People**	**%**	**95%CI**	
A/H1N1/Michigan	Before vaccination	20/32	63	[43-79]	2/6	33	[4-78]	*p* = 0.22
	After 3 weeks	27/30	90	[73-98]	2/6	33	[4-78]	***p*** **= 0.008**
	After 3 months	8/9	89	[52-100]	3/6	50	[12-88]	*p* = 0.24
Dynamics analysis^b^		***p***^**1−0**^ **= 0.04**, *p*^3−1^ = 1.00			*p*^1−0^ = 1.00, *p*^3−1^ = 1.00			-
A/H3N2/Singapore	Before vaccination	22/32	69	[50-84]	2/6	33	[4-78]	*p* = 0.17
	After 3 weeks	29/30	97	[83-100]	3/6	50	[12-88]	***p*** **= 0.01**
	After 3 months	7/9	78	[40-97]	4/6	67	[22-96]	*p* = 1.00
Dynamics analysis		***p***^**1−0**^ **= 0.02**, *p*^3−1^ = 0.50			*p*^1−0^ = 1.00, *p*^3−1^ = 1.00			-
B/Colorado	Before vaccination	7/31	23	[10-41]	0/6	0	[0–46]	*p* = 0.57
	After 3 weeks	19/29	66	[46-82]	0/6	0	[0-46]	***p*** **= 0.005**
	After 3 months	6/9	67	[30-93]	0/6	0	[0-46]	***p*** **= 0.03**
Dynamics analysis		***p***^**1−0**^ **= 0.002**, *p*^3−1^ = 1.00			*p*^1−0^ = 1.00, *p*^3−1^ = 1.00			**-**
B/Phuket	Before vaccination	13/31	42	[25-61]	0/6	0	[0–46]	*p* = 0.07
	After 3 weeks	25/29	86	[68-96]	0/6	0	[0-46]	***p*** **< 0.001**
	After 3 months	7/9	78	[30-93]	0/6	0	[0-46]	***p*** **= 0.007**
Dynamics analysis		***p***^**1−0**^ **< 0.001**, *p*^3−1^ = 1.00			*p*^1−0^ = 1.00, *p*^3−1^ = 1.00			-

a *The exact Fisher test was used*,

b *the McNemar test with the Holm-Bonferroni correction was used, p-value^1−0^, p-value^3−1^–the statistical significance of the difference between the control point of 3 weeks and the initial level and between the control points of 3 months and 3 weeks, respectively*.

*Differences in seroprotection levels to strains A/H1N1 and A/H3N2 between groups of healthy control and CVID patients were observed 3 weeks after vaccination. Seroprotection levels to strains B/Colorado and B/Phuket between these groups statistically significant differed both 3 weeks and 3 months after vaccination*.

*Bold values indicate seroprotection reference levelis over 70%*.

In the group of healthy participants, a statistically significant increase in the proportion of seropositive was observed 3 weeks after immunization toward all strains. As a result, seroprotection level to strains A/H1N1, A/H3N2, and B/Phuket meets the criterion of CHMP effectiveness (at least 70%) and is 90, 97, and 86%, respectively. Seroprotection level to strain B/Colorado is 66% that is close to the threshold value. 3 months after vaccination in the group of healthy participants the seroprotection level remains the same or slightly lower than achieved a month after immunization; statistically significant decrease was not detected for any strain.

The proportion of seropositive in the group of CVID patients did not change statistically significant after vaccination, remaining at the level of 0% to strains B/Colorado and B/Phuket, 33–50% to strain A/H1N1 and 33–67% to strain A/H3N2.

Initially, before vaccination, regardless of the strain, the proportion of seropositive between groups of healthy control and CVID patients did not statistically significant differ. Differences in seroprotection levels to strains A/H1N1 and A/H3N2 between groups of healthy control and CVID patients were observed 3 weeks after vaccination. Seroprotection levels to strains B/Colorado and B/Phuket between these groups statistically significant differed both 3 weeks and 3 months after vaccination. Probably no differences were detected after 3 months, due to a slight increase in proportion of seropositive among CVID patients and a small amount of them.

### Seroconversion Level

**Figure 2:**
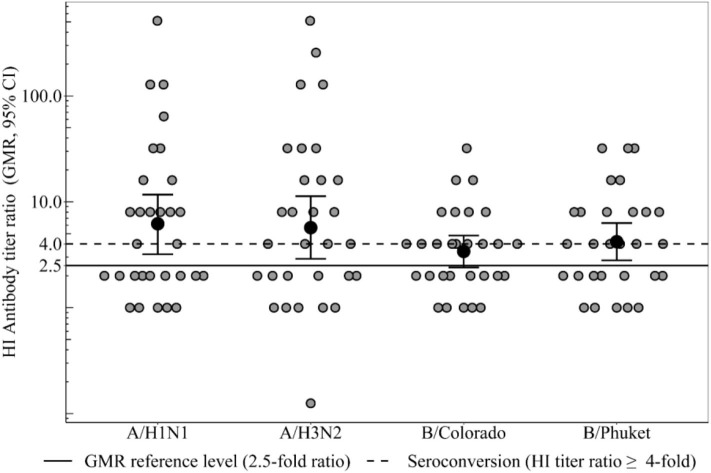
HI antibody titers ratio (3 weeks after immunization), which show the ratio of individual titers of healthy participants and GMR (95% CI) in the group of healthy participants. Seroconversion level in the group of healthy participants 3 weeks after immunization to all vaccine influenza virus strains met the CHMP criterion (not <40%). GMR in the group of healthy participants for all 4 strains meets the CHMP criterion of effectiveness (at least 2.5). In the group of patients with CVID GMR did not reach the threshold minimum for any strain.

Seroconversion level in the group of healthy participants 3 weeks after immunization (Figure 2) to strain A/H1N1 was 50% [95%CI = [31–69%], 15 participants out of 30], to strain A/H3N2−60% [95%CI = [41–77%], 18 out of 30], to strain B/Colorado−45% (95%CI = [26–64%], 13 out of 29], to strain B/Phuket−52% [95%CI = [33–71%], 15 out of 29] that meets the CHMP criterion (not <40%). In the group of CVID patients seroconversion level (Table 2) was 0% to all strains except strain A/H3N2−17% (one person out of six). Thus, statistically significant differences in the level of seroconversion were revealed between groups of healthy and CVID patients regardless of the virus strain.

**Table 2:** Individual HI antibody titers, seroconversion level and GMR in the group of CVID patients.

**Strains**	**Patients**	**HI antibody titers from individual patient**			**HI Antibody titers ratio (3 weeks)**
		**Before**	**3 weeks**	**3 months**	
A/H1N1	1	20	10	10	0.5
	2	20	20	20	1
	3	40	40	40	1
	4	20	20	20	1
	5	20	20	40	1
	6	80	40	40	0.5
	Seroconversion level (3 weeks): 0%, 95%CI = [0–46%], *p* = 0.03—compared to healthy^a^				
	GMR (3 weeks): 0.8, 95%CI = [0.5–1.2], *p* < 0.001—compared to healthy^b^				
A/H3N2	1	10	5	5	0.5
	2	5	5	20	1
	3	40	40	40	1
	4	10	80	40	8
	5	20	20	40	1
	6	80	80	40	1
	Seroconversion level (3 weeks): 17%, 95% CI = [0–64%], *p* = 0.05—compared to healthy				
	GMR (3 weeks): 1.3, 95% CI = [0.5–3.4], *p* = 0.014—compared to healthy				
B/Colorado	1	5	5	5	1
	2	5	5	5	1
	3	10	10	20	1
	4	5	10	20	2
	5	5	5	10	1
	6	20	10	10	0.5
	Seroconversion level (3 weeks): 0%, 95% CI = [0–46%], *p* = 0.04—compared to healthy				
	GMR (3 weeks): 1.0, 95% CI = [0.6–1.6], *p* = 0.002—compared to healthy				
B/Phuket	1	10	5	5	0.5
	2	10	10	10	1
	3	20	10	10	0.5
	4	5	5	10	1
	5	10	10	10	1
	6	20	20	20	1
	Seroconversion level (3 weeks): 0%, 95% CI = [0–46%], *p* = 0.03—compared to healthy				
	GMR (3 weeks): 0.8, 95% CI = [0.5–1.2], *p* < 0.001—compared to healthy				

a *The exact Fisher test was used*,

b *the Mann-Whitney test was used*.

### Geometric Mean Rate

GMR in the group of healthy participants (Figure 2) for all four strains meets the CHMP criterion of effectiveness (at least 2.5) and amounts to strain A/H1N1 6.2, to strain A/H3N2 5.7, to strain B/Colorado 3.4 and to strain B/Phuket 4.2. In the group of patients with CVID GMR did not reach the threshold minimum for any strain. Thus, GMR is statistically significant higher in the group of healthy participants compared with CVID patients regardless of the virus strain (Table 2).

### Geometric Mean Antibody Titers

GMT in the groups of healthy and CVID patients are presented in the Table 3 and Figure 1.

**Table 3:** GMT in the groups of healthy participants and CVID patients.

**Strains**	**Period**	**Healthy participants**		**Patients with CVID**		**Between groups^a^**
		**GMT**	**95%CI**	**GMT**	**95%CI**	
A/H1N1	Before vaccination	35.9	[20.4–63.1]	28.3	[15.4–52.0]	*p* = 0.72
	After 3 weeks	237.0	[119.8–468.7]	22.4	[13.0–38.8]	***p*** **< 0.001**
	After 3 months	296.3	[66.5–1319.7]	25.2	[13.9–45.6]	***p*** **= 0.01**
	Dynamics analysis ^b^^,^^c^	***p***^**1−0**^ **< 0.001**, *p*^3−1^ = 0.09		*p*^1−0^ = 0.17, *p*^3−1^ = 0.36		–
A/H3N2	Before vaccination	41.8	[24.9–70.2]	17.8	[6.1–52.0]	*p* = 0.18
	After 3 weeks	266.0	[155.1–456.1]	22.4	[5.9–85.3]	***p*** **< 0.001**
	After 3 months	186.8	[35.5–972.6]	25.0	[10.4–60.3]	***p*** **= 0.05**
	Dynamics analysis	***p***^**1−0**^ **< 0.001**, *p*^3−1^ = 0.08		*p*^1−0^ = 0.57, *p*^3−1^ = 0.74		–
B/Colorado	Before vaccination	13.1	[8.4–20.3]	7.1	[3.8–13.0]	*p* = 0.07
	After 3 weeks	45.2	[26.6–76.7]	7.1	[4.8–10.5]	***p*** **< 0.001**
	After 3 months	42.9	[10.7–174.2]	10.0	[5.5–19.1]	***p*** **= 0.05**
	Dynamics analysis	***p*^1−0^ < 0.001**, *p*^3−1^ = 0.07		*p*^1−0^ = 1.00, *p*^3−1^ = 0.08		–
B/Phuket	Before vaccination	21.4	[13.7–33.3]	11.2	[6.5–19.4]	*p* = 0.21
	After 3 weeks	94.6	[58.1–153.8]	8.9	[5.2–15.4]	***p*** **< 0.001**
	After 3 months	86.4	[25.2–296.7]	10.0	[6.3–15.8]	***p*** **= 0.007**
	Dynamics analysis	***p***^**1−0**^ **< 0.001**, *p*^3−1^ = 0.07		*p*^1−0^ = 0.17, *p*^3−1^ = 0.36		–

a *Student criterion was used*,

b *Student criterion was used for paired samples*,

c *all criteria were counted on pre-logarithmized data*.

*The groups of healthy and CVID patients differ statistically significant throughout the post-vaccination period, regardless of any strain*.

*Bold values indicate seroprotection reference levelis over 70%*.

*p-values ^1−0^, ^3−1^ the statistical significance of the difference between the control point of 3 weeks and the initial level and between the control points of 3 months and 3 weeks, respectively*.

In the group of healthy participants a 3 weeks after immunization statistically significant increase in antibody titer was observed for all virus strains. Three months after vaccination the GMT of antibodies remains unchanged relatively to the 3 weeks level.

In the group of CVID patients the GMT remains unchanged throughout the whole period, regardless of any strain. As a result the groups of healthy and CVID patients differ statistically significant throughout the post-vaccination period, regardless of any strain.

## Discussion

### What Is Known About Adjuvanted TIV (aTIV)?

Currently, the use of the trivalent adjuvant vaccine against influenza virus in the world is proved in people ≥65 years old compared with QIV and non-adjuvant TIV in accordance with the statement by the Public Health England and Joint Committee on Vaccination and Immunization (25).

Monovalent A(H1N1) influenza vaccine with adjuvant AS03 (Pandemrix®, GlaxoSmithKline, Belgium) was widely used during a pandemic 2009–2010 influenza season in order to form a specific immune response in a short time, both among a healthy adult population and immunocompromised patients (boosted after 1 month), in whom it showed encouraging results.

The European adjuvanted influenza vaccine with MF59C.1® which is composed of 9.75 mg squalene showed significant immunogenicity. Moreover, several meta-analyses have shown a statistical superiority of aTIV, independently from the (sub)type considered, and high immunogenicity against drifted/heterologous strains, especially against A(H3N2) (26).

In numerous investigations conducted in Russia, it was shown that the immunogenicity and protective properties of antigens, attached to the synthetic high molecular weight polymer carrier—azoximer bromide, increase tens of times, enhance both antibody and cell-mediated immune responses, enlarges synthesis of all classes of protective antibodies (IgM, IgG, IgA), except IgE (27). Thus, it is a strong activator of B- and T-lymphocytes and this finding has led for subsequent clinical use in various groups of patients with abnormalities in immune system, as well as for the production of influenza vaccines.

The first adjuvanted influenza vaccine (Grippol) in Russia was introduced into healthcare practice in 1997 and contained in addition to 500 μg of azoximer bromide 5 μg of hemagglutinin each of the influenza viruses type A (A/H1N1 and A/H3N2) and 11 μg of type B (one lineage) influenza virus. Then in 2008 Grippol Plus vaccine was registered with a reduced number of antigens of the influenza virus type B from 11 to 5 μg without losing its immunogenic properties.

At the stage of clinical registration studies among adults of Grippol plus and the tetravalent vaccine (Grippol Quadrivalent—appeared in Russia in 2018), containing 500 μg of azoximer bromide in addition to 5 μg of hemagglutinin from each of 3 or 4 strains of influenza A and B viruses, respectively, their accordance with the immunogenicity criteria for inactivated influenza vaccines was proved for all strains: for TIV the level of seroprotection was 76–95%; in seronegative individuals, the level of seroconversion reached 73–95%, the geometric mean rate (GMR) 6.7–23.6 (28–30); for QIV 1 month after vaccination of healthy volunteers, the seroconversion level to strains A/H1N1, A/H3N2, and B/Yamagata, B/Victoria was 65.8, 69.3, 65.8, and 67.8%, respectively, and the geometric mean rate (GMR)−4.9, 5.3, 5.4, and 4.8 respectively.

Numerous post-registration studies conducted in 2009–2019 were devoted to assessing the safety, immunogenicity, prophylactic and clinical efficacy of the trivalent polymer subunit vaccine against influenza in different risk groups, such as: pregnant women (31–38), elderly people aged 60 and over with cardiovascular system diseases (39, 40), children and adults with asthma and other chronic obstructive respiratory tract diseases (41–44), that showed high immunogenicity and good tolerance in all participants from risk groups who are subject to vaccination as part of the Russian national immunization program. Even in patients ≥60 years with diseases of cardiovascular system the level of seroconversion was 49.5–68.5%, GMR was 2.8–5.7, and seroprotection was 83.7–84.8%.

### What Is Known About QIV?

According to the results of numerous investigations carried out at the clinical stages of QIV studies and after their use in practice, non-inferiority of antibody responses to QIV comparing with TIV for the matched strains, and its superiority for not corresponding B strain was shown (9). In two phase-III clinical trials totally among 6,224 adult volunteers aged ≥18 years (4,659 in one study and 1,565 in another) in comparison with the TIV, the QIV displayed superior immunogenicity toward the alternative-lineage B strain, without impairing the immune responses to shared strains. Moreover, QIV vaccines proved similar reactogenicity and safety (45, 46).

Some models for influenza virus type B spread in the world were elaborated. According to Eichner et al. with a retrospective analysis over the past 50 years of usage TIV 11.2% of cases type B influenza infection could have been prevented using QIV (8). According to the results of another study during the period 2000–2013 QIV would prevent, on average, about 16% more cases of type B influenza infection than TIV when the vaccine and circulating strains do not match, suggesting that cross-protection is 70% between B lines. It has also been shown that old people (≥65 years old) and adults aged 50–64 years benefited the most from QIV, with a decrease of 21 and 18% of infections, respectively (47). Depending on the levels of vaccine-induced cross-protection between B lineages decrease in cross-protection to 50, 30, and 0% effectiveness of QIV increases up to 25, 30, and 34% relatively B lineage infections.

### Vaccination in Patients With CVID

In patients with CVID, belonging to the group of immunocompromised patients, vaccination, despite the regular administration of donor immunoglobulins, is the only way to form a protection against the virus, as well as to prevent infectious complications of influenza. At the same time, there is a study reporting the content of cross-reactive A/H1N1 antibodies in IVIG (48). However, in a study by Gardulf et al. in 48 patients with CVID, despite regular IVIG therapy (1 time per week), this fact is not confirmed (20).

Only this study and the study by Pedersen et al. [2/3 patients with CVID showed protective level of antibodies with a >4-fold increase by seroconversion after double dose of the vaccine at the beginning and boostarization after 3 weeks (7.5 + 3.75 μg)] have presented data from the use of the specific adjuvant X179a in individuals with CVID whereas in healthy population in investigations it has been shown that 67–98.3% produce protective levels of antibodies against the influenza A(H1N1) 21days after injection of a single dose (3.75 μg) of the vaccine Pandemrix® (49, 50).

While Eibl and Wolf believe that vaccination against the influenza virus should follow the same schemes as in healthy individuals (51), data from clinical studies show the need for boosting dose not earlier than 21 days after vaccination, or the introduction of a double dose at the same time with the goal of more active stimulation (19, 20).

In our study we decided to vaccinate patients with CVID with 1 dose of the tetravalent adjuvanted influenza vaccine on the background of absence of IVIG immunotherapy for 7 weeks. An immune response was expected due to the presence of an adjuvant that activates innate immunity factors, as well as an expanded spectrum of antigens in the vaccine, in contrast to the study using the Pandemrix mono-vaccine.

### Our Results

In healthy patients vaccinated with a tetravalent adjuvant vaccine with a reduced amount of antigens against all 4 strain-specific surface antigens up to 5 μg, this vaccine has proved its immunogenicity in such criteria as seroprotection (≥70%), seroconversion (≥40%), GMT, and GMR (≥2.5). The level of seroconversion to strain A/H3N2 is statistically significantly higher in the group of initially seronegative (in 2.5 times: 100 vs. 40%).

Based on an individual analysis of the specific antibodies titer in each patient with CVID, it can be noted that 3 out of 6 patients showed ≥2-fold increase in the titer of antibodies to the influenza type B/Victoria lineage (moreover, in 1/6 the antibody growth was 4 times) 3 months after vaccination, that had not been observed in blood sample analysis after 3 weeks. This may indicate the need for a study of post-vaccination antibodies to various infections in such patients not earlier than 4 weeks after immunization. However, protective antibody titer of ≥1:40 to strains of two lines (Yamagata and Victoria) was not achieved at all and was no more than 1:20. Although it should be noted that for patients with CVID, a conditional indicator of the effectiveness of vaccination is an 2–4-fold increase of post-vaccination antibody level in relation to the pre-vaccination.

As for the level of antibodies to A/H3N2, three patients showed an increase in antibody titer to 1:20, 1:40, and 1:80 (by 2, 4, and 8 times respectively); they were also found in 2/6 in blood samples 3 months and in 1/6–3 weeks after immunization.

For A/H1N1 strain only one patient in the post-vaccination period showed an increase in antibody titer by 2 times after 3 months compared with the initial (1:20) and reached a protective level (1:40).

Therefore, the obtained results on the assessment of post-vaccination immunity using a quadrivalent immuno-adjuvant vaccine indicate that in 50% of patients (3/6) with CVID an increase in antibody titer to strain A/H3N2 is observed (at protective level in 2/6 patients, 33.3%); to A/H1N1- in 16.7% (1/6 patients) at the protective level; to B/Victoria—in 50% (3/6 patients) there is an increase in antibody titer without reaching a protective level; to B/Yamagata—increase in titers was not detected.

Considering the fact that only one patient out of 6 with CVID showed a protective level of antibodies in the control blood sample 3 weeks after vaccination, while the rest of the patients had antibodies only 3 months later, we can suggest that patients with CVID should probably conduct a follow-up analysis for greater reliability no earlier than 4 weeks after immunization.

The search for new vaccination schemes is the subject of further investigations, as well as the effectiveness of boosterization with immunoadjuvant vaccines in patients with CVID.

## Conclusion

A detailed assessment of the immunogenicity of a tetravalent influenza virus vaccine with a reduced concentration of antigens of all four strains with the inclusion of an immunoadjuvant in healthy was made, its immunogenicity similar to that of the non-adjuvant QIV in the world. In patients with CVID the use of this vaccine was also investigated for the first time, and encouraging results were obtained.

## Data Availability Statement

All datasets generated for this study are included in the article.

## Ethics Statement

The studies involving human participants were reviewed and approved by Ethical Committee of National Research Center—Institute of Immunology Federal Medical-Biological Agency of Russia. The patients/participants provided their written informed consent to participate in this study.

## Author Contributions

AK: investigation and writing—original draft. NA: methodology, resources, and data curation. EL: conceptualization and writing—review & editing. AV: visualization and formal analysis. YD and SK: investigation. TL: writing—review & editing. MK: methodology, resources, and project administration. All authors contributed to the article and approved the submitted version.

## Conflict of Interest

The authors declare that the research was conducted in the absence of any commercial or financial relationships that could be construed as a potential conflict of interest.
